# School-Based Obesity Prevention Intervention in Chilean Children: Effective in Controlling, but not Reducing Obesity

**DOI:** 10.1155/2014/618293

**Published:** 2014-04-27

**Authors:** Juliana Kain, Fernando Concha, Lorena Moreno, Bárbara Leyton

**Affiliations:** Institute of Nutrition and Food Technology (INTA), University of Chile, Santiago, Chile

## Abstract

*Objective*. To evaluate the effectiveness of a 12-month multicomponent obesity prevention intervention. * Setting*. 9 elementary schools in Santiago, Chile. * Subjects*. 6–8 y old low-income children (*N* = 1474). * Design*. Randomized controlled study; 5 intervention/4 control schools. We trained teachers to deliver nutrition contents and improve the quality of PE classes. We determined % healthy snacks brought from home, children's nutrition knowledge, nutritional status, duration of PE classes, and % time in moderate/vigorous activity (MVA). Effectiveness was determined by comparing Δ BMI *Z* between intervention and control children using PROCMIXED. * Results*. % obesity increased in boys from both types of schools and in girls from control schools, while decreasing in girls from intervention schools (all nonsignificant). % class time in MVA declined (24.5–16.2) while remaining unchanged (24.8–23.7%) in classes conducted by untrained and trained teachers, respectively. In boys, BMI *Z* declined (1.33–1.24) and increased (1.22–1.35) in intervention and control schools, respectively. In girls, BMI *Z* remained unchanged in intervention schools, while increasing significantly in control schools (0.91–1.06, *P* = 0.024). Interaction group ∗ time was significant for boys (*P* < 0.0001) and girls (*P* = 0.004). * Conclusions*. This intervention was effective in controlling obesity, but not preventing it. Even though impact was small, results showed that when no intervention is implemented, obesity increases.

## 1. Introduction 

Obesity in Chile is considered the most important public health problem in children. Over the last decades the prevalence of obesity has tripled and is presently 23% in 6 y old children [1]. The government and private sector have undertaken several initiatives over the last 10 years, but although some interventions (especially school-based) have shown to be effective while they are in place, the net result has been disappointing [2].

The significant rise in childhood obesity in Chile can be attributed to the environmental factors shown in both developed and developing countries as being associated with this condition, except that, in some developing countries such as Chile, these risk factors have penetrated in a much shorter period of time than in other countries where economic progress has been slower [2]. It has been reported that not only chronic diseases are the main causes of morbidity and mortality in low and middle-income countries, but approximately 80% of all chronic disease burden occurs in these countries [3], so prevention is of utmost importance.

Because targeting dietary intake and physical activity in school settings are probably the most popular form of addressing childhood obesity, school-based interventions have been implemented in all countries where this condition constitutes a public health problem. Since 2002, we have addressed childhood obesity by developing, implementing, and evaluating school-based prevention programs. We implemented a pilot study and a 2-y controlled intervention including 6–12 y old children in a small city close to Santiago called Casablanca. We then implemented a pilot study and a 2-y controlled intervention in a district of Santiago called Macul for 4–9 y old students which included a wellness program for their teachers. Both interventions consisted in training teachers to deliver contents on healthy eating and increasing physical education classes and additionally, in Macul, teachers participated in a wellness program. BMI *Z* score and obesity prevalence were compared between children in intervention and control schools by year and between students of intervened and control teachers. In the Casablanca study, impact was greatest on the younger children, during the first school year when the study was fully resourced. In Macul, although intervened teachers exhibited improvements in anthropometry and blood measures, impact on the children was not related to their results [2]. After the intervention in Macul, the educational authorities of Ñuñoa, also a district located in Santiago, asked us to implement and evaluate an obesity prevention program for students attending public primary schools in the district.

The objective of this paper is to report the effectiveness of the multicomponent primary prevention program applied during 12 months for 1st to 3rd grade children from public schools in Ñuñoa. The intervention included classroom nutrition education, increasing physical education (PE) class time, and increasing time children were moderately active during those classes.

## 2. Methodology

### 2.1. Participants

There are 10 primary public schools in Ñuñoa; of these, we excluded one, because in 2010 one of our students had carried out a pilot program in that school. In 2011, we selected the sample for this intervention; it included children from kindergarten to 2nd grade from the 9 schools. They were followed during 12 months (4 in 2011 and 8 in 2012). The total sample size amounted to 1471 children. We assessed the post hoc power and significance that results for a sample size of 1500 subjects. Power was estimated considering that, over one year, BMI *Z* score would increase by 0.3 [6] with a power of 80% and significance of 0.05. This calculation resulted in 400 children in each group, intervention and control.

### 2.2. Randomization

This is a cluster-randomized study in which schools were randomized into intervention and control as follows. Public schools in Chile are beneficiaries of the School Food Program, which includes the provision of free breakfast and lunch. The number of food rations that each school is entitled to receive is based on what is called the “school's vulnerability index,” which is calculated by an entity belonging to the Ministry of Education at the central level [7]. This index considers socioeconomic (SES) and anthropometric variables determined on children attending 1st grade and has been shown to be a proxy of the SES of the students [8]. Based on this index, we categorized the schools into 3 groups resulting into 2, 3, and 4 schools from the lowest to the highest SES, respectively. Randomization to intervention and control was done within those categories resulting in 5 and 4 intervention and control schools, respectively. Of the 1474 children who attended 1st to 3rd grade in 2012, 651 were intervention and 823 controls. Although the control group included more children, because the school with significantly more students was randomized into this group, the sample size was sufficient to detect changes over the entire period, as explained before.

INTA's Ethical Committee approved the program. In addition, parents received information on the intervention and had to approve a written consent to allow their children to participate in the study.

### 2.3. Study Design

We began the second semester of 2011 with measurements, training of teachers, and limited parent involvement, while in 2012 we continued with these components and evaluated the intervention. We report in this paper the process and effectiveness of the program after 12 months (4 months in 2011 and 8 in 2012).

### 2.4. Intervention

Table 1 summarizes the main components of the program in 2011 and 2012. The primary outcome measures were BMI *Z* score and obesity prevalence, while secondary outcomes were children's knowledge on healthy eating, types of foods brought to school, and degree of implementation by teachers of the educational contents on healthy eating and physical education.

### 2.5. Data Analysis

#### 2.5.1. Nutrition Education

Classroom education consisted of a brief theoretical part and practical work in the form of activities like painting and puzzles. Teachers in intervention schools were evaluated by the study nutritionist in terms of how well they applied the nutrition contents by comparing the results of their mean scores during the first versus the second semester. This score was obtained by adding the results obtained on 6 aspects: teacher mentions objectives, gives clear instructions, knows the topic, moves around the class to check what the children are doing, and summarizes the most important points. Each item had a maximum of 3 points, so the perfect score was 18 points. We used *t*-test to compare the mean scores by semester.

To determine if changes in children's knowledge of healthy eating in intervention and control schools were different, we compared the delta (Δ) % of correct answers between baseline and followup in each group, using the test of proportions.

In 43 classes (out of a total of 55) of children attending 1°, 2nd, and 3rd grades, whose mean ages were 6.6, 7, 7, and 8.9 y, respectively, we compared the % of “healthy snacks” children brought from home one day in April and one in October 2012 and also compared the difference in the Δ of this proportion between intervention and control children, using the test of proportions

#### 2.5.2. Anthropometry

With the weight and height of the children we calculated BMI and BMI *Z* score using the WHO reference [9]. Low weight was defined as BMI *Z* ≤ −1, normal weight as BMI *Z* between –1 and + 1, overweight as BMI *Z* between +1 and +2, and obese as BMI.

Consider *Z* > +2. Nutritional status and BMI *Z* score were calculated in August 2011 (baseline) and November 2012 (followup) and the results were compared. 1949 children had data on weight and height at baseline and 1669 had it only at followup, while 1474 had both (76.6% of the original sample). It is important to note that, of those measured at followup, 214 children were new students who began the school year in March 2012, so they were not present when we measured at baseline. To test if the BMI of children lost to followup was different from that of children included in the sample, we compared their mean BMI *Z* at baseline; these figures were 1.08 (1.13) and 1.12 (1.2), respectively, with no significant difference (*P* = 0.52).

#### 2.5.3. Physical Education Classes

We observed 36 and 15 PE classes during the 1st semester and 56 and 32 classes during the 2nd semester of 2012 in intervention and control schools, respectively, and compared the change in the median duration of classes from the first to the second semester between trained and untrained teachers (using Wilcoxon test) in classes observed at least 4 times each semester (*N* = 18 and 12 in intervention and control schools, resp.). In addition, in those same PE classes, we also collected data on the following curricular aspects related to how the class was conducted: teacher explains clearly the objectives, teacher is motivated, degree of preparation of the class, and if the structure of the class follows the objectives. Each of these 4 items ranked from 1 (worse) to 4 (best), so the total score had a maximum of 16 points. We compared the change in the median score obtained by PE teachers from the first to the second semester between intervention and control teachers using the Wilcoxon test.

Finally, in 8 of those classes (4 each semester) we compared the Δ% of class time children engaged in moderate and vigorous activity (MVA) between intervention and control children, using the test of proportions. The data on MVA was collected from pedometers (New Life Style 1000) placed on every child (right hip) during the class in intervention and control children.

#### 2.5.4. Effectiveness

We compared BMI *Z* score and % obesity between baseline and followup by school using *t*-test and the test of proportions, respectively, considering *P* < 0.05 as significant. In addition, we compared the same variables as well as % overweight by sex.

The effectiveness of the intervention was determined by comparing changes in BMI *Z* score of intervention and control children for each sex between baseline and followup using mixed model of covariance (PROC MIXED, SAS) and the interaction group ∗ time, adjusting for baseline value (the interaction was considered significant if *P* < 0.05). The Tukey test was used post hoc by sex, to test if changes within each group and between groups over time were significant.

## 3. Results 

We compared the mean score obtained by trained teachers each semester for the 6 aspects regarding how they implemented the nutrition contents. Of a maximum of 18 points, the mean score was 13.7 and 14.7 the 1st and 2nd semester, respectively, that is, 76 and 81.5% (this change was not significant). The item with the lowest score was “knows the topic” followed by “summarizes the main points.”

Ninety percent of children 1st to 3rd grade brought snacks to school; these were generally a combination of cookies or crackers with juice. The % of healthy food items brought by children in intervention and control schools was 33 and 38% in April and 46.3 and 38% in October, respectively, a significant difference in intervention children during the year as well as the Δ change between them (*P* = 0.016).

The mean % of correct answers regarding healthy eating of children in 1st grade in 2011 was 73 and 74% in intervention and control schools, respectively. At followup (2nd grade in 2012) these figures were 92 and 87%. The same analysis for children in 2nd grade in 2011 gave the following results: 45 and 32% and 57 and 33% in intervention and control schools, respectively. In both grades and type of schools, there was a significant increase in the % of correct answers. No difference between these deltas was observed for children in 1st grade and an almost significant increase (*P* = 0.0057) among children in 2nd grade 2011 from intervention schools.

Table 2 shows the change in BMI *Z* score and % obesity of the children by school. BMI *Z* declined significantly in children from one intervention school; it stayed unchanged in children from the other intervention schools. In children from 3 out of 4 control schools, BMI *Z* increased significantly. The prevalence of obesity remained unchanged in children from intervention schools while increasing in children from control schools; however, in only one of them, this increase was significant.

Table 3 (boys) and Table 4 (girls) show the change in the prevalence of overweight and obesity between baseline and followup. The prevalence of overweight increased slightly in boys from control schools, while the opposite occurred in boys from intervention schools. The % of obese boys increased nonsignificantly in both types of schools. In girls (Table 4), the prevalence of overweight increased nonsignificantly in girls from both types of schools; however this increase was greater in those from intervention schools. The prevalence of obesity among girls in control schools increased by 3.7 percentage points, while decreasing slightly in intervention schools; however these changes were not significant.

Table 5 shows the changes in the quality of PE classes (90-minute class time) conducted by trained versus untrained teachers in terms of real class duration, curricular aspects of the class, and % time children engaged in MVA during actual class time. The median actual class time was around 60 minutes during the first semester, increasing nonsignificantly to 64.8 and 67.9 minutes in classes conducted by trained and untrained PE teachers, respectively. There was a significant increase in the Δ change of the median score obtained related to class implementation by trained teachers (from 10.1 to 14.3). Minutes of MVA and consequently % of class time children engaged in MVA were very low and declined in classes conducted by untrained teachers (24.5 to 16.2% of MVA) while remaining unchanged in classes conducted by trained teachers (24.8 and 23.7%).

**Figure 1:**
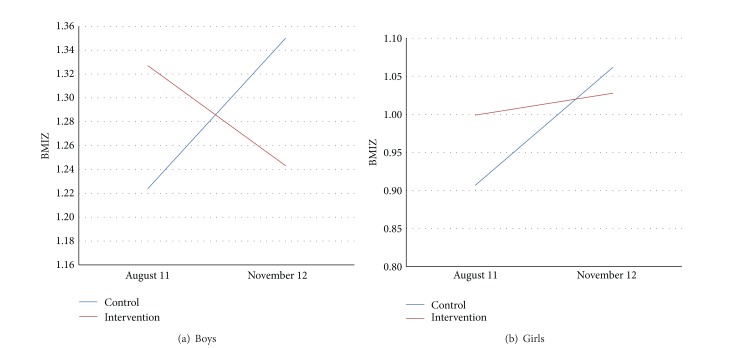


Figure 1 shows the change in BMI *Z* from baseline to followup and its interaction in boys (Figure 1(a)) and girls (Figure 1(b)) from intervention and control schools. In boys from intervention schools, BMI *Z* declined slightly (nonsignificantly from 1.33 to 1.24), while in those from control schools the opposite was observed (BMI *Z* increased from 1.22 to 1.35). In girls from intervention schools, BMI *Z* remained unchanged, while increasing significantly in those from control schools (0.91 to 1.06, *P* = 0.024). The PROC MIXED analysis showed that the group ∗ time interaction was significant for both boys (*P* < 0.0001) and girls (*P* = 0.004).

## 4. Discussion 

This multicomponent intervention included a set of activities related to healthy eating and physical activity as part of a wider program. It is important to point out that specifically these activities (and not others) were implemented because school principals and teachers only accepted the implementation and evaluation of the ones we report here. The only curricular initiative consisted in extending PE class time, while the others included training classroom teachers to deliver contents on healthy eating and PE teachers to improve the quality of their classes.

The main result of this study shows that this intervention was effective in controlling the rise in overall BMI *Z*, while the prevalence of obesity (the upper tail of the curve) increased nonsignificantly in boys and slightly decreased in girls. Although the effectiveness of the intervention was small, it stopped the rise in BMI *Z* observed over the years [10].

Some positive results were obtained by variables measuring process. The greatest improvement was observed on the type of snacks children brought to school (almost 50% brought only healthy foods at followup compared to 38% in control schools). Although the increase in knowledge on healthy eating was slightly higher in children exposed to the intervention, nutrition education is considered crucial in obtaining behaviour change leading to dietary improvements [11, 12].

Significant improvements on aspects related to how PE teachers conduct their classes were observed. We used pedometers to determine the amount of time at which children engage in MVA as this was our outcome variable for quality of PE classes. Tudor-Locke and Lutes [13] in their search for evidence on using pedometers for assessing physical activity concluded that these devices correlate strongly with different accelerometers, with the highest agreement during walking and running. Even though in our study the time at which children engaged in MVA remained low and unchanged in PE classes conducted by trained teachers, it declined significantly in those from control schools. Other authors have found even lower % time in MVA during PE classes. Simons-Morton et al. [14], in a study including 157 fifth-grade students from Texas that determined the quality of PE classes (40 minutes per class) conducted by specialists, found that on average children spent only 8.5% of class time in MVA, considerably less than what we found.

In contrast, other authors have found significantly better results. Because PE classes can contribute significantly to the total daily proportion of MVA (as much as 50.4% of total daily MVA) as reported by Raustorp et al. [15], the quality of the PE class should be considered as a very important aspect to be addressed in increasing total daily MVA.

As stated by the American Academy of Pediatrics [16], without maintaining or improving program quality, additional physical education time could be wasted.

In a review published in 2010 on Latin American school-based physical education programs [17], the authors found that only 5 studies met the inclusion criteria; two of them dealt with PE classes. This review reported that physical activity increased from 5% to 69% for moderate and 50% to 55% for vigorous activity. In contrast, we found that % time in MVA remained unchanged in PE classes conducted by trained teachers and declined significantly at followup in control schools.

A recent Cochrane review of 26 school-based physical education studies showed some positive impact on the duration of physical activity and the proportion of children that engage in MVA during school hours, while having a limited impact on BMI [18]. Considering the high rates of childhood obesity in most Latin American countries [19], there is an urgent need to develop, implement, and evaluate school-based PE programs.

There have been numerous multicomponent school-based interventions to address rising obesity rates. Recently,* Lekker Fit*! in the Netherlands [20] was developed mainly to focus on the increase of the quality and amount of PA of primary school children, classroom education on healthy eating, and active lifestyle. At the one-year followup, results showed significant intervention effects on the prevalence of overweight, waist circumference, and fitness of 6–9 year old children; however no effect was found on BMI. Another study which found an effect on the prevalence of overweight and not on BMI [21] was the El Paso Catch Study in Texas, which included a minimum of three PE lessons a week as well as a classroom curriculum on multiple health behaviours. In contrast to those findings, we found an effect on BMI, as this intervention specifically targeted children with a BMI *Z* > 2. When we compared the mean BMI *Z* at baseline and followup in children whose baseline BMI *Z* was between 1.5 and 2, we obtained 1.74 (0.14) and 1.82 (0.52) for children in control schools and 1.71 (0.14) and 1.71 (0.47) for those in intervention schools. The same analysis for children with a baseline BMI *Z* > 2 gave 2.74 (0.64) and 2.71 (0.66) for children in control schools and 2.81 (0.86) and 2.54 (0.8) for children in intervention schools. A *t*-test of the differences in BMI *Z* revealed no significant change in BMI *Z* for those with a baseline BMI *Z* between 1.5 and 2 (*P* = 0.24) and a significant decline in BMI *Z* for children in intervention schools whose baseline BMI *Z* was >2 (*P* < 0.0001) (not shown). These results show that the intervention mostly targeted the very obese children.

The latest meta-analysis on prevention of childhood obesity [22], which included 37 studies of 27,946 children, demonstrated that in general programs were effective at reducing adiposity with a high level of observed heterogeneity among the results. Overall, children in the intervention group had a standardized mean difference BMI *Z* of −0.15 (95% CI: −0.21 to −0.09). Intervention effects by age subgroups were −0.26 (95% CI: −0.53 to 0.00) (0–5 years) and −0.15 (95% CI −0.23 to −0.08) (6–12 years). The authors state that heterogeneity was apparent in all age groups and could not be explained by randomization status or the type, duration, or setting of the intervention. Our intervention was less effective as the overall change in BMI *Z* was only −0.035, (95% CI −0.03 to −0.04); however in the control schools BMI *Z* increased by 0.14 (95% CI +0.13 to +0.15). Of all the components found by this meta-analysis to contribute to the beneficial effects observed, we were able to address the “school curriculum that includes healthy eating, physical activity and increased sessions for physical activity and support teachers to implement health promotion strategies.” There was limited parental support as this initiative only addressed types of recommended snacks brought to school while we could not change the nutritional quality of the food sold in the schools.

The strengths of our study are the use of a cluster-randomized design, a relatively large sample size, and the objective measures of the primary and secondary outcomes.

There are several limitations. We were not able to implement two activities that were programmed. The first one was greater parental involvement, a key aspect in achieving changes in dietary intake and physical activity of their children [23]. We aimed at participating once a month in motivational sessions with most parents for around 45 minutes; however only around 40% of parents attend regular meetings. In addition, we were allowed to participate 3 times and for only 15 minutes. So, on average our overall annual contact with parents was 45 minutes which we used to underscore the types and combination of healthy snacks and ask them not to give money to their children to buy from the school kiosk (fortunately only 3% of the children carried money to school). The second initiative was the transformation of the school kiosk into one that offers 80% of healthy foods. Each school in Chile has at least one kiosk which is “rented” to someone with the condition that he (she) pays a monthly rent (around US $ 200). The money is used for certain basic things such as paper and photocopies. These kiosks generally sell packaged high calorie foods and a small proportion of “healthy foods.” These practices are in sharp contrast with the nutrition knowledge students receive in the classroom and contribute to unhealthy dietary habits. In intervention schools, we trained in 2 sessions (4 hours in total) “kiosk owners” in what types of foods they could sell in order to gradually have a “healthy kiosk,” which we define as offering 80% of healthy foods. After they complained that due to lower sales they would not be able to pay the “rent,” school principals did not back this initiative. Schools in other countries have also been shown to rely on profits to support different school activities. In the US, for example, some schools can generate considerable revenue [24], have negotiated contracts for products sales, and even obtain money in return for selling exclusively some foods. In Chile, kiosks in public schools generally do not earn a sizable profit and do not hold contracts with food or beverage companies. Fortunately, a law has been passed and hopefully will be applied in 2014 that kiosks will be able to sell competitive foods that comply with predefined nutritional standards.

Lastly, apart from extending PE class time, the educational aspect of the intervention, that is nutrition and physical activity, are not part of the curriculum, so unless they are not included, the probability of the program being sustainable in the future is low. School engagement is critical in this.

In conclusion, our results provide evidence that this 12-month multicomponent intervention to prevent obesity in 6–8 y old children was effective in controlling obesity, but not preventing it, specifically targeting those with the highest BMI *Z* score. Even though this outcome may seem limited, results from children in control schools show that if no intervention is in place, obesity continues to rise as demonstrated by the school annual census of the nutritional status of children in 1st grade [10].

## Figures and Tables

**Table 1:** Brief description of the main components of the intervention (2011-2012).

August–November 2011	March–November 2012
*Data collection* (1) Anthropometry: weight and height of all children kindergarten-2nd grade (*N* = 1474), mean age, 6.6 (1.07) y with trained and standardized nutritionists. (2) Children's knowledge on healthy eating (1st and 2nd grades) Validated questionnaire including 8 items based on figures *N* = 266 children *Training in 5 intervention schools* (1) Two nutritionists trained teachers from kindergarten-3rd grade on the correct application of the contents of a special booklet [4] that includes 8 sessions of 90 min each on health eating for the children (6 hrs of training) *N* = 38 teachers (2) Teachers of PE classes from 1st*–*3rd grade were trained (6 hrs) by a specialist on the use of a book. [5] containing a leaflet for each class which includes drawings of different exercises recommended to increase MVA (*N* = 12 teachers) (3) Training of kiosk owners (4 hrs) using a book which shows how to gradually offer 80% of healthy foods (*N* = 8 owners).	*Data collection: children 1st–3rd grade or mean age 7.7 *(*1.2*)* y* (1) In 43 classes (from a total of 55) we collected, during one day in April and one in October, data on the types of foods children brought to school Data collected had to be anonymous (*N* = 592 and 431 children in intervention and control schools, respectively, in April and 577 and 411 in October) (2) We assessed 2 aspects of PE classes during each semester: effective class time and 4 curricular aspects related to the class (3) We determined the % of time children engaged in MVA with pedometers placed on all children during 8 PE class (*N* = 482 and 155 intervention and control children, resp.) (4) We determined during 8 visits how well teachers in intervention schools implemented the nutrition education program (*N* = 25 teachers) (5) We repeated the application of the questionnaire measuring children's knowledge on healthy eating (6) We collected the weight and height of the children that were evaluated 14 months before
*Parents * During one regular school meeting, the study nutritionist briefly explained in every class the objectives of the program and specifically the types and combination of snacks considered to be “healthy”	*Training teachers of children 1st–3rd grade* At the beginning of the school year we repeated the training process for newly hired teachers (3 teachers for healthy eating and 1 for physical education (6 hrs each) *Parents of children 1st–3rd grade* Twice during the year in each class, the study nutritionist briefly interacted with parents (15 minutes)

In 2011 there were 2 weekly PE classes, one lasting 90 minutes and the other one 45 minutes. In 2012, the duration of the second class increased to 90 minutes. Control schools followed the regular curriculum.	

**Table 2:** Comparison of BMI *Z* score and prevalence of obesity by school (2011-2012).

	Sample Size	BMI *Z*			% obesity		
		August 2011	November 2012	*P *	August 2011	November 2012	*P *
Intervention schools							
Brigida	56	0.77 (1.7)	0.91 (1.33)	0.326	23.2	23.1	0.99
B. Claro	114	1.45 (1.1)	1.27 (1.1)	0.0002	28.1	25.4	0.83
E. Frei	249	1.14 (1.1)	1.12 (1.1)	0.7	22.1	22.6	0.43
C. Rica	170	1.14 (1.14)	1.13 (1.14)	0.814	24.0	25.1	0.95
R. Francia	58	1.4 (1.4)	1.31 (1.37)	0.264	26.3	25.4	0.82
Control schools							
G. Zañartu	112	1.22 (1.2)	1.34 (1.2)	0.08	21.4	25.4	0.27
J. Toribio	176	1.11 (1.2)	1.39 (1.1)	0.0001	22.2	29.4^1^	0.0003
Lenka	117	1.11 (1.3)	1.26 (1.3)	0.0035	26.5	29.9	0.46
Siria	416	0.99 (1.1)	1.1 (1.1)	<0.001	16.8	18.5	0.32

**Table 3:** Change in overweight and obesity prevalence between boys in control and intervention schools (2011-2012).

Variables	Control		Intervention	
	*N* = 423		*N* = 364	
	August 2011	November 2012	August 2011	November 2012
Age (y)	6.7 (1.1)	8 (1.1)	6.5 (1.1)	7.9 (1.1)
BMI (kg/m^2^)	17.7 (2.6)	18.7 (3)	17.80 (2.5)	18.3 (2.7)
% overweight	28.4	30.0	29.4	27.2
% obesity	26.3	29.5	26.6	28.3

**Table 4:** Change in overweight and obesity prevalence between girls in control and intervention schools (2011-2012).

Variables	Control		Intervention	
	*N* = 400		*N* = 287	
	August 2011	November 2012	August 2011	November 2012
Age (y)	6.7 (1)	7.9 (1)	6.5 (1)	7.8 (1)
BMI (kg/m^2^)	17.4 (2.3)	18.3 (2.6)	17.6 (2.6)	18.3 (2.9)
% overweight	33.8	34.5	26.6	32.6
% obesity	13.3	17.0	21	18.8

**Table 5:** Change in characteristics of PE classes conducted by teachers in control and intervention schools*.

	Control children		Intervention children	
Period of observation of PE classes of children 1st–3rd grades	May–July 2012	Aug.–Oct. 2012	May–July 2012	Aug.–Oct. 2012
Number of PE classes observed at least 4 times each semester	12	12	18	18
Actual duration of PE classes (median minutes)	61.4	64.8	58.8	67.9
Four curricular aspects related to the way the teacher conducts the class (median score of a total of 16 points)	9.9	10	10.1	14.3^1^
Number of children wearing pedometers in 4 PE classes each semester	155	155	482	482
Moderate/vigorous activity (MVA)^a^ in PE classes: mean minutes (SD)	13.8 (5.6)	10.2 (5.6)^2^	15.6 (5.3)	16.1 (5.0)
% Class time in MVA (calculated from real duration)	24.5	16.2^2^	24.8	23.7

*All PE classes had a programmed duration of 90 minutes; ^a^moderate and vigorous activity.

^1^ *P* = 0.033; ^2^ *P* = 0.000.
